# The role of androgen therapy in acquired aplastic anemia and other bone marrow failure syndromes

**DOI:** 10.3389/fonc.2023.1135160

**Published:** 2023-05-08

**Authors:** Momen Nassani, Riad El Fakih, Jakob Passweg, Simone Cesaro, Hazzaa Alzahrani, Ali Alahmari, Carmem Bonfim, Raheel Iftikhar, Amal Albeihany, Constantijn Halkes, Syed Osman Ahmed, Carlo Dufour, Mahmoud Aljurf

**Affiliations:** ^1^Department of Hematology, Stem Cell Transplant & Cellular Therapy, King Faisal Specialist Hospital and Research Center, Riyadh, Saudi Arabia; ^2^Department of Hematology, University Hospital Basel, Basel, Switzerland; ^3^Pediatric Hematology Oncology, Azienda Ospedaliera Universitaria Integrata, Verona, Italy; ^4^Transplantation Unit, Department of Hematology, Hospital de Clinicas, Federal University of Parana, Curitiba, Brazil; ^5^Department of Hematology, Armed Forces Bone Marrow Transplant Centre, Rawalpindi, Pakistan; ^6^Department of Hematology, Leiden University Medical Center, Leiden, Netherlands; ^7^Hematology Unit, Hemato.Oncology Department, IRCCS, G. Gaslini Children Research Institute, Genova, Italy

**Keywords:** androgen, aplastic anemia (AA), bone marrow failure (BMF), hematopoiesis, anabolic steroid

## Abstract

Bone marrow failure syndromes are a heterogeneous group of diseases. With the major advancements in diagnostic tools and sequencing techniques, these diseases may be better classified and therapies may be further tailored. Androgens, a historic group of drugs, were found to stimulate hematopoiesis by enhancing the responsiveness of progenitors. These agents have been used for decades to treat different forms of bone marrow failure. With the availability of more effective pathways to treat BMF, androgens are less used currently. Nevertheless, this group of drugs may serve BMF patients where standard therapy is contraindicated or not available. In this article, we review the published literature addressing the use of androgens in BMF patients and we make recommendations on how to best use this class of drugs within the current therapeutic landscape.

## Introduction

Aplastic anemia (AA) is a term used to describe a group of heterogeneous syndromes that affect hematopoiesis and result in bone marrow failure (1). It can be inherited, acquired or a result of exposure to certain toxins. This syndrome was first described by the German physician Paul Ehrlich in 1885 (1). Back then, it used to be a fatal condition due to uncontrolled bleeding or overwhelming infections. The advancement in allogeneic stem cell transplantation and immunosuppressive treatment in the 20th century changed the outcome of the disease dramatically (2, 3). The long term overall survival of bone marrow failure (BMF) syndromes have remarkably improved in the modern era and can exceed 90% (4). Trials comparing transplant to conventional therapies (including androgens) showed clear survival benefit of transplant compared to conventional therapies (5, 6). Nevertheless, limitations and challenges in the management of aplastic anemia and BMF still exist. Allogeneic stem cell transplant may not be feasible for all patients for a number of reasons, especially in countries with limited resources. Alternatives to transplantation include immunosuppressive therapy, growth factors, thrombopoietin agonists and androgen therapy. The objective of this article is to provide a comprehensive and systemic evaluation of the published literature regarding the use of androgens in acquired and to a lesser extent inherited bone marrow failure syndromes.

## History of androgen use in aplastic anemia

In the early days, the management of AA patients consisted of supportive transfusions in addition to other interventions with questionable efficacy (steroids, splenectomy, vitamins, etc.) (7). The first indications about the possible efficacy of androgens in bone marrow failure, were the spontaneous remission in two boys upon pubescence and the development of myeloid metaplasia in a patient taking testosterone for breast cancer (7). A small report followed, where five AA patients who failed steroids and transfusion were given testosterone and four of them achieved remarkable responses in hemoglobin levels and transfusion independence along with variable response in neutrophils and platelets (7). A number of publications were reported thereafter supporting the role of testosterone and anabolic steroids in aplastic anemia (8–11). Overall, around 70% of patients on these studies achieved a hemoglobin above 12 g/dl along with improvement in the platelet and neutrophil numbers. Another multicenter trial recruited 45 patients with hypoproliferative or aregenerative anemia treated with oxymetholone for a minimum of 3 months. Patients with hypocellular marrow had the best response (12). In 1976, a prospective randomized clinical trial showed superiority of allogeneic bone marrow transplantation compared to standard of care, which included androgen therapy (5). A clear reduction in mortality was reported in the transplanted group compared to the non-transplant group. Currently, with the consistent encouraging results of transplant over the last decades, HSCT is considered the standard of care for young, fit SAA patients with available donors. Other trials compared different compounds of anabolic steroids. One of these trials, showed better outcome with methandrostenolone compared to other types of anabolic steroids, while methanolone was associated with the worst response and survival rates in this study (13). In another study, where 125 patients with AA were randomized to receive four different androgens (norethandrolone 1 mg/kg/day, fluoxymesterone 1 mg/kg/day, stanozolol 1mg/kg/day and testosterone undecanoate 1.7 mg/kg/day) fluoxymesterone treated group had the best overall survival. The worst survival rate was in patients on stanozolol (14).

## Testosterone and anabolic androgenic steroids

The body building action of androgens and their euphoric action on the brain led to widespread illicit use of AAS. Hence, all AAS were designated as class III controlled substances. Nevertheless, these agents have shown significant benefits in a number of disorders. Testosterone was first discovered in 1935 and found to have effects on both reproductive (androgenic) and non-reproductive tissues (anabolic). It has been used in different catabolic states due to its anabolic effects through nitrogen fixation and as such protein synthesis (15). Virilization, on the other hand, is one of the unwanted side effects. Numerous derivatives have been developed aiming to prolong its biological activity, increase its anabolic effects and decrease the androgenic side effects. These derivatives are commonly known as anabolic androgenic steroids (AAS) (15). More than a hundred synthetic products have been developed by different reactions (17 α-alkylation, 17 β-esterification, etc.) to overcome the rapid biotransformation of testosterone and synthesize orally active longer acting compounds (9, 15). Oxandrolone, oxymetholone and nandrolone are commonly used AAS, whereas danazol (2,3-isoxazol-17α-ethynyltestosterone) is a synthetic steroid with antiestrogenic, antigonadotropic, and androgenic activities (15). Hepatotoxicity is a potential side effect of anabolic steroids traditionally observed with the 17 α-alkylated compounds (methyltestosterone, oxymetholone, oxandrolone, norethandrolone, etc.) (16). Around a quarter of patients may experience elevation in liver function tests while on therapy and liver tumors are not uncommon (9). Early studies reported fatality cases from liver disease and jaundice; however, it is unclear if these were due to the anabolic steroids or other potential complications observed in patients with BMF like viral hepatitis, iron overload. The optimal recommended dose of androgens is not well defined; however, it is well known that patients who do not respond to a certain dose may achieve remission using a higher dose of the same product. The recommended doses of the commonly used products are: 2.5 mg/kg/d for oxymetholone and methanolone and 1 mg/kg/d for methandrostenolone and Norethandrolone (9, 13). Given the biological effects of AAS, serious adverse events can happen (masculinization, aggression, liver dysfunction and adenomas among others). Close medical supervision and dose adjustment to the minimal effective dose is recommended. Androgens should be avoided in pregnant women, cancer patients (prostate, breast, etc.), patients with nephrotic syndrome or liver dysfunction and patients with hypercalcemia of malignancy. The androgen side effects (flushing, acne, hirsutism, change in voice, others) usually disappear quickly after discontinuation (17). **Table 1** is a summary of side effects and authors’ recommendations on how to mitigate these.

**Table 1:** Potential side effects of androgens and authors’ recommendations on how to mitigate these.

Side effect	Comments	Mitigation plan
Virilization, Gain of body musculature	Testosterone causes masculinization, flushing of skin and acne, deepening and hoarsening of voice, changes in external genitalia	Use the smallest effective dose whenever possible
Jaundice, hepatotoxicity, hepatoma, hepatocellular carcinoma, Peliosis hepatis	Peliosis hepatis: blood filled enlarged sinusoids and cysts focally or throughout the liver	Monitor Liver enzymes every 3 months Initial Screen for hepatitis B, C, Ferritin Initial screening by liver US and every 6 months thereafter Avoid concurrent hepatotoxic medications Consider Using non 17 alpha alkylated androgens
Polycythemia	Monitor hemoglobin	Slow tapering of androgens once normal hemoglobin is achieved
Hyperlipidemia	Lipid profile return to normal within one month after stopping androgens	Dietary advice to avoid excessive fat intake Avoid other cardiovascular risk factors Exercise Consider statin
Psychiatric and behavioral effects	Monitor for psychiatric symptoms	Use the smallest effective dose whenever possible

## Immunosuppression and androgens therapy in aplastic anemia

A randomized trial compared anti-lymphocyte globulin (ALG) (with androgens and haploidentical HSCT vs. Androgens alone) showed 76% vs. 31% survival respectively at two years (p <0.002) (18). Another trial randomized 121 patients to receive anti-thymocyte globulin (ATG) alone or ATG with androgens showed similar response (44% vs 42% respectively) and survival rates (19). In a subsequent trial, 15 patients received ATG and methanolone and 15 patients received ATG alone (20). The response rate was 73% in the combination group with eight complete responses compared to 33% in the ATG alone group with two complete responses (P = 0.01). The difference in survival (87% in the combination arm vs 43%) was not statistically significant. Shahidi et al. treated 23 AA patients with oxymetholone and cyclosporine combination. Thirteen of these patients had already received ATG and did not respond and the remaining 10 did not receive ATG. The response rate was 38% and 70% respectively (21). Further, a randomized controlled trial showed significant difference in responses among males and females to androgens, where females with low neutrophil counts had significant benefit from ATG combined with androgens compared with ATG alone (22). Bacigalupo et al. randomized 134 patients to ALG and methylprednisolone with or without oxymetholone. At 4 months, the response rate with significantly higher in patients who received oxymetholone (56% vs 40%; P < 0.04) (22). In a relatively recent report, Jaime-Pérez et al. reported the outcomes of fifty AA patients (23). Thirteen patients were transplanted and 37 patients were not eligible to transplant, had no access to IST, and as such were treated with danazol (median dose 400 mg) and supportive measures. The five-year OS was in favor of the transplant group (92% vs 41%, p = 0.001). The ORR in the danazol group was 46% with a median time to respond of 3 months. Although, transplant and IST are highly efficacious in AA, androgens continue to be an option, even in the frontline, for transplant ineligible patients with no access to modern IST.

## Androgens for other BMF syndromes

Congenital bone marrow failure syndromes are a heterogeneous group of cytopenias associated with various congenital defects and cancer predisposition. HSCT is the only curative treatment for the hematological complications related to these diseases, but many patients are ineligible and androgens are considered the main non-transplant modality to treat these patients. Early studies of using androgens in these patients showed favorable responses (8). Oxymetholone and danazol are frequently used for Fanconi anemia (FA) and dyskeratosis congenita (DC) patients with responses reaching up to 80% (24–26). Diamond-Blackfan anemia patients are usually treated with steroids but many of them fail steroids (tolerance, side effects, relapse) and eventually receive androgens (24). Oxymetholone is the androgen of choice for congenital anemias with a starting dose of 0.5-2 mg/kg/day. Expectedly, response starts within 4-8 weeks and once a hemoglobin concentration of 12 g/dl is reached the dose is reduced gradually to the minimum effective dose to maintain hemoglobin between 10-12 g/dl (25). In paroxysmal nocturnal hemoglobinuria (PNH), a rare acquired clonal stem cell disorder, androgens were efficacious to treat the anemia part of the disease especially in patients with hypoplasia (27–29). However, androgens have no effect on hemolysis and their impact on thrombogenesis need to be watched closely in these patients.

## Fanconi anemia

Fanconi anemia is the prototype of inherited bone marrow failure syndromes characterized by a number of mutations leading to genetic instability and multiple cancer susceptibility with frequent incidence of marrow failure and myelodysplasia (30–32). Clinically, FA patients are characterized by diverse congenital malformations (small stature, skeletal malformations, hyperpigmentation, urogenital abnormalities, etc.) (32). In a small cohort of FA patients, seven out of eight patients responded to danazol 5 mg/kg/day and had stable counts up to 3 years (33). In another cohort, seven out nine patients treated with oxandrolone had a hematologic response with major side effects being elevated liver function tests and virilzation (34). A retrospective trial analyzed 70 patients who received androgens for FA. Out of 70 patients, 37 were evaluable. Oxymetholone was the most frequently used androgen. Hematologic response was seen in 25 out 37 evaluable patients (68%) with a median of 6.5 g/dl improvement in hemoglobin (median time to respond of 14 weeks), a median of 70000 platelet count increase (median time to respond of 11.5 weeks) and a median of 1350/ml improvement in neutrophils (median time to respond of 12 weeks) (35). Virilization, liver toxicity, liver adenomas and clonal evolution were the most frequently reported adverse events. In the largest retrospective trial addressing androgen use in FA patients, 66 patients were reported; 49 received oxymetholone and 17 received danazol (36). Danazol was started at a dose of 2-4 mg/kg/day and oxymetholone was started at as dose of 0.5-1 mg/kg/day. After a median duration of therapy of 18 months, 52 patients (78%) achieved hematologic response and 30 patients (45%) had trilineage response. There was no difference in response rates between danazol and oxymetholone. Seven patients (11%) developed grade 3 liver toxicity that was noticed more in patients on oxymetholone. Peliosis hepatis developed in one patient on oxymetholone. The majority of patients developed virilzation signs. These reports included patients from different age groups (age range: 3 – 22 years).

## Androgens for telomeropathies

Telomeres are essential for genomic stability but their length decreases with each cellular division. Dyskeratosis congenita (DC) is the prototype of telomeropathies. Androgens improve blood counts and reduce transfusion frequency in telomeropathies. Some studies have shown improvement in telomere length as well (37–41), although other reports did not confirm this finding (42). Apparently, the improvement in telomere length depends on the underlying mutational profile (41). In a phase 1/2 study, danazol (800 mg/day) was administered to patients with telomeropathies. In 12 evaluable patients, telomere elongation was achieved in all. Hematologic response was seen in 19 of 24 evaluable patients (79%) after 3 months of therapy. Liver toxicity (41%) and muscle cramps (33%) were the most frequently reported side effects (37). In another study, 26 DC patients were followed prospectively. Ten patients received androgens and 16 were not treated. There was no statistical difference in telomere length in the two groups (42). Seven out of the 10 treated patients had a consistent RBC and platelets response to androgens. In a more recent report, seven patients were treated with androgens (danazol, oxymetholone). All patients had hematological response as well as significant increase in telomere length (41). An ongoing trial (clinicaltrials.gov NCT02055456) is evaluating nandrolone decanoate (parenteral androgen with no first hepatic pass) in patients with telomeropathies. These reports included patients from different age groups (age range: 3 – 66 years). **Table 2** is a summary of androgen studies in AA and BMF syndromes.

**Table 2:** Important studies about androgens in BMF patients.

Author and date	Number of patients	Disease	Androgen	Response	Comments
Camitta 1983 (18)	13	Severe acquired aplastic anemia	Oxymetholone vs. Antithoracic duct lymphocyte globulin (ATDLG)	31% OS in androgen arm Vs. 76% in ATG group	ATG compared to androgen
Champlin R 1985 (19)	52	Moderate to severe Aplastic anemia	ATG+Androgen (oral androgen oxymetholone or fluoxymesterone) Vs ATG+placebo	ATG+Androgen ORR 42% ATG+Placebo ORR 44% P:>0.9 OS: 55 vs 50% P:.65	
Kaltwasser J 1988 (20)	30	Aplastic anemia	ATG with or without oral androgen (Methenolone)	ATG+androgen ORR73% ATG alone ORR =31% P=0.01 OS=87% vs 43% p=0.15	
Shahidi N 1990 (21)	23	Aplastic anemia	oxymetholone and cyclosporine	Post ATG failure ORR: 38% Not exposed to ATG ORR 70%	
Bacigalupo A 1993 (22)	134 (69/65)	Acquired aplastic anemia	HALG + methylprednisolone with or without oxymetholone (RCT)	ORR: 68% vs. 48% For females: 78% vs. 27%	RCT Response to androgen is more prominent in females with low ANC
Shahidi NT 1961 (8)	7	Inherited aplastic anemia Fanconi anemia	Testosterone + steroid	Increase reticulocytosis 7/7 Transfusion independence 6/7	Most patients needed to stay on low dose therapy
Scheckenbach K 2012 (33)	8	Fanconi anemia	Danazol	ORR: 7/8	
Rose SR 2014 (34)	9	Fanconi Anemia	Oxandrolone	ORR 7/9	
Paustian L 2016 (35)	37	Fanconi anemia	Oxymetholone Danazol Methonolone Enanthate Norethandrolone	ORR: 68%	Median time to response 14 weeks
Ribeiro L 2015 (36)	66	Fanconi anemia	Oxymetholone, danazol	ORR 78%	
Townsley DM 2016 (37)	27	telomere diseases	Danazol	ORR 79%	All evaluable 12 patients had a gain in telomere length at 24 months as compared with baseline
HARTMANN RC 1966 (28)	6	PNH	flyoxymesterone	ORR 5/6	Improved anemia in responders with persistent low grade hemolysis
Halder R 2020 (29)	20 pediatric patients	Classical PNH anemia with hemolysis, normal platelets and neutrophils	Danazol or stanazolol	ORR 80% CR 30%	
Jaime-Pérez 2011 (23)	50 patients	Aplastic anemia	Danazol Vs Allogenic transplantation	ORR in danazol group =46% OS (92% vs 41%, p = 0.001). in favor of transplant	
Payal P. Khincha 2018 (42)	10 patients treated with androgen vs 16 untreated	dyskeratosis congenita	oxymetholone danazol, halotestin	ORR 7/10	There was no statistical difference in telomere length among two groups
Martin Kirschner 2021 (41)	7 patients	dyskeratosis congenita	danazol, oxymetholone	ORR 7/7	All patients had hematological response as well as significant increase in telomere length

## Mechanism of action and effects on hematopoiesis

The exact mechanism of action of androgens in stimulating hematopoiesis remains unknown, however it seems that the use of supra-physiological doses of androgens cause erythropoiesis expansion. This is in contrast to hematinic supplements (iron, folate) where excess supply will not cause excessive response in hematopoiesis. Earlier studies showed that androgens enhance the responsiveness of erythroid progenitors to erythropoietin and possibly enhance the growth of pluripotent and committed granulocyte/macrophage progenitors (19). Some of the anabolic steroids have unique properties, for example, danazol inhibits interleukin-1 and TNF-α production (a property of corticosteroids) and has myelosuppressive effects (43). More recent studies showed that androgens stimulate erythropoietin production and release, activation of the erythropoietin receptor on progenitor cells and increase iron incorporation into the red cells (44, 45). Additionally, androgens increase telomerase activity in hematopoietic cells (39). Lately, a study showed that the chronic use of oxymetholone improves hematological parameters by diminution of quiescence and promotion of proliferation of hematopoietic progenitors and stem cells. Oxymetholone down regulated the transcription of osteopontin, a cytokine that up-regulates the expression of certain interferons and interleukins, which inhibits cellular proliferation. Hence, it was proposed that oxymetholone suppression of osteopontin transcription induces hematopoietic stem cell cycling (46). The earliest phase of erythroid response is characterized by erythrocytosis that happens shortly after initiating androgen therapy followed by delayed improvement in hemoglobin. This delay is probably related to an initial increase in erythrocyte destruction while patients are still transfusion dependent. Usually, the improvement of hemoglobin may be observed as early as 3 months or as late as 6 months after starting androgen therapy (13). The increase in neutrophils usually mirrors the response of hemoglobin. The response rate of neutrophils was found to be 35.8% in one case series (8, 13). Platelets are the latest to improve and usually show less prominent improvement (13). The response rate of platelets is observed in about one-third of cases. In one study, normal platelets were achieved in 18 out of 67 patients (8, 13). Of note that despite the minimal improvement in the numbers of neutrophils and platelets, the clinical benefit, in terms of less bleeding and infections is disproportionately higher (9). Responding patients can achieve a normal hemoglobin level and a protective platelets and neutrophils levels (8). The bone marrow examination of patients receiving androgens show groups of growing stromal cells, reduction of fat cells and the appearance of erythroid hyperplasia foci. These changes are usually seen within 3 months of treatment initiation (7). In summary, response to androgens starts 3 to 6 months after treatment initiation and responding patients may enjoy durable response in around 50% of cases while the other 50% will relapse with relapse being higher in rapidly tapered patients (< 3 months) (9, 17). A second remission can be achieved with re-treatment but these patients will need continuous androgen therapy thereafter (17). Upon withdrawal of androgens, the reticulocytes and hemoglobin drop within the first month, and then stabilize during the second month with similar effects in the neutrophils and platelets.

## Practical author’s recommendations and clinical scenarios

The diagnostic armamentarium is expanding with the use of new and more comprehensive sequencing and molecular techniques. These new diagnostic tools help to better delineate these syndromes and to identify more patients with cryptic alterations who may not benefit from immunosuppression. A number of innovative ideas and approaches (gene therapy, leucine, quercetin, etc.) to address the unmet needs of these patients are in ongoing trials. Bone marrow failure patients who are not candidates for transplant (elderly, comorbidities, no donors, etc.) and patients with the non-severe forms are usually defaulted to non-transplant medical interventions. In affluent countries, a number of non-transplant options are available for this group of patients. However, in restricted resources countries these novel options are not readily available. Androgens are considered a classic old group of medications that can stimulate hematopoiesis and as such used for this group of disorders when modern resources are not available or have failed already. Androgens result in hematologic responses (transient in some patients however) in most patients with FA and telomeropathies but does not alter nor affect the risk of clonal evolution. A number of pros and cons have to be considered when a decision is made to treat a BMF patient with non-transplant modalities including the use of androgens. Factors linked to a potential better response to androgens include higher residual cellularity, mild to moderate cytopenia, toxin induced BMF, women with lower absolute neutrophil count, the use of higher dose of androgens and rapid improvement of counts after androgen initiation (9, 13, 14). The presence of a number of these factors in a patient may sway the managing team to consider androgens, while the presence of contraindications may push the team to consider alternatives. Below are selected scenarios in which the authors’ believe androgen therapy should be considered.

- As a bridge to transplant in patients with symptomatic FA and telomeropathies
- Older SAA patients, not candidate for HSCT and failed standard immunosuppression therapy (IST) and thrombopoietin mimetic (TPO) or TPO inaccessible
- Patients with renal failure precluding the use of calcineurin inhibitors and failed TPO mimetic or TPO inaccessible
- Multiply relapsed patients after failing standard lines of therapy
- PNH patients with no access to complement inhibitors and parallel existing AA (AA/PNH overlap)

Currently, there are no published trials looking into the safety, efficacy and different dosages of various AAS formulations to guide the clinicians’ choice when treating BMF patients. New trials looking into these issues are warranted.

## Author contributions

MA, RE, and MN designed and performed the research. MN wrote the first draft. All authors contributed to the article and approved the submitted version.
